# South West Orthopoedic Club—Meeting in Truro, April 1988

**Published:** 1989-08

**Authors:** 


### South West Orthopaedic Club

### Meeting at the Royal Cornwall Hospital, Treliske, Truro, April 1988

**A meeting of the South West Orthopaedic Club was held at the Royal Cornwall Hospital, Treliske on the 23rd April. It was the fortieth anniversary of the Club making it the oldest of the regional Orthopaedic Clubs and at the beginning of the afternoon session the Honorary Secretary read a short paper on the events which lead to the first meeting and included a description of the Minutes of the first meeting.**

**At the end of the meeting Mr Robert Robins, Senior Orthopaedic Surgeon at Truro gave a talk entitled 'Robert Jones and the Colonial Frontier', which reviewed the history of orthopaedics in Cornwall.**

**Mr G Bannister of the Southmead Hospital, Bristol was the Gary Hampson Memorial Lecturer and after an introduction by Mr Tony Ratliff he delivered a lecture entitled 'The fixation of the femoral component in total hip replacement.'**

### SYNOVECTOMY OF THE KNEE IN RHEUMATOID ARTHRITIS

**P M Hutchins, Truro**

In view of the continuing controversy as to the value of synovectomy in rheumatoid arthritis, a retrospective review of cases performed in Edinburgh with a minimal 10 year follow-up was performed. Of 154 patients with a total of 194 knees, 75 patients with 100 knees were traced and examined. Forty patients, with 47 knees, were lost to follow-up and 39 patients had died.

Most patients had persistent swelling and pain, despite an adequate pre-op period of medical treatment. In all patients, synovectomy was performed through a single medial parapatella incision aided by meniscectomy in most cases.

At 10 year follow-up, 39% of the knees had failed, having come to, or were awaiting further surgery. Of the survivors only 10% had significant pain, 48 could walk more than 1 kilometre and 70% used no walking aid. Soft tissue swelling persisted in 40% and flexion to more than 90° was present in 69% of cases.

### PATELLAR SUBLUXATION FOLLOWING KINEMATIC KNEE ARTHROPLASTY 'IS THE UNIVERSAL INSTRUMENT SYSTEM UNIVERSAL?'

**D P Johnson, Bristol**

Following total knee arthroplasty the incidence of patello-femoral subluxation has been reported to be up to 30%. Excessive pre-operative or post-operative valgus or external rotation has been proposed as the cause. The kinematic knee prosthesis was designed and first proposed as being inserted with the components aligned perpendicular to the mechanical axis of the leg. However, since 1981 it has been suggested that the implant should be inserted with the 'Universal' instruments, placing the components perpendicular to the vertical axis during one legged stance, at 3° of valgus to the mechanical axis. A prospective study was performed of 56 patients undergoing kinematic knee arthroplasty to determine if this valgus position increased patello-femoral subluxation.

Subluxation or dislocation of the patella occured in 13 cases (24%), in 6 it was accompanied by pain which was often disabling. The patients with subluxed patellae had less valgus and external rotation deformities than patients with normal patello-femoral tracking ($p<0.05$). None of the 8 patients in which a lateral release was performed sustained a subluxed patella. It was also determined that the 'Universal' instruments incorrectly shape the femur, in the antero-posterior size, and the peg fixation holes which have to be drilled in two positions. The patello-femoral subluxation is not attributable to valgus or external rotation deformities. Implants inserted with the 'Universal' instrumentation should incorporate measures to prevent patello-femoral subluxation and therefore be reserved for the PCA knee for which they were designed.

### COMPUTED TOMOGRAPHY AS THE RADIOLOGICAL INVESTIGATION OF BACKACHE IN A DISTRICT GENERAL HOSPITAL

**R B Sellwood, W H Smith, C R Howie, Royal Cornwall Hospital, Truro**

The impact of computerised tomography in suspected disc disease or stenosis of the lumbar spine has been examined in a district general hospital, 462 scans in 462 patients were reviewed. Seventy patients who had CT evidence of disc prolapse underwent surgery. In 86% of these cases the radiological diagnosis was confirmed.

A fall in the number of myelograms from 12 per month to three per month was documented and 84% of lumbar disc surgery carried out during the last year of review was undertaken without myelography.

We believe that where disc prolapse is suspected on the history and clinical examination that a CT scan should be the radiographic investigation after plain radiographs. Further, if the scan confirms the clincial suspicion of disc prolapse then a myelogram is unnecessary.

### FOOT PRESSURE PLATE ANALYSIS AS AN INDICATOR OF SUCCESS IN FOREFOOT SURGERY

**Marcellino Maheson, Cardiff**

A prospective trial which included all patients undergoing forefoot surgery throughout the calendar year 1985 were assessed pre-operatively by myself clinically, and radiologically and the analysis of the way distribution of the forefoot as assessed by the pressure plate.

One year later, all patients were reviewed and re-assessed clinically, radiologically and pressure plate analysis re-done. Only 16% of patients were satisfied with the results of their surgery and in these the pressure plate analysis showed that the weight bearing through the hallux and first metatarsal was maintained. 84% of patients were dissatisfied with their surgery because their pain was unchanged or more severe than originally and that it had moved further laterally, in the forefoot.

Pressure plate analysis revealed in these patients that the hallux and first metatarsal were either not or minimally involved in weight bearing and the majority of the weight was taken in the second, third and fourth metatarsals and toes.

This work shows that the aim of forefoot surgery should be to keep the hallux and first metatarals as the primary weight bearing area and that surgical success can then be more accurately expected.

### THE EFFECT OF SPINAL ANAESTHESIA ON URINARY FUNCTION AND BLOOD LOSS IN TOTAL HIP ARTHROPLASTY

**Mr G A Gie, Dr C Collins, Princess Elizabeth Orthopaedic Hospital, Wonford Road, Exeter**

This prospective study presents a series of 157 consecutive total hip arthroplasties in which patients were randomly allocated into two groups.

1) Light general anaesthetic with spinal anaesthesia.
2) General anaesthetic without spinal anaesthesia.

All the operations were performed by a single surgeon (GAG) using an extended posterior approach. Marcaine, with or without Diamorphine, was used routinely for the spinal anaesthetic. Intra- and post-operative blood loss was closely monitored as was the ability to pass urine post-operatively.

84 patients (52 females and 32 males) were given a spinal anaesthetic and 73 patients (41 females and 32 males) were not. Of those females who had a spinal, 11.5% required catheterisation as compared to 4.9% of those without spinal. In the males, 31.25% went into retentaion (21.9% requiring catheterisation) as compared to 12.5% in the non-spinal group. Intra-operative blood loss was 20% lower in the spinal group. No patient has yet presented with deep sepsis with a follow-up ranging from 7 to 20 months.

### MAYO'S ARTHROPLASTY—? A FORGOTTON PROCEDURE

**R S Majkowski, B D A Morris, Cheltenham General Hospital**

An operation for the treatment of Hallux Valgus first des cribed by Charles Mayo eighty years ago has since been compared with other proceedures involving Excision Arthroplasty of the first Metatarsophalyngeal joint in a number of studies. In the authors original description he emphasised the importance of narrowing the foot if the deformity was not to recur. This parameter does not seem to have been measured in any of these studies. Today Mayo' Procedure seems to have fallen into relative disfavour. Thi study looks at the results of the modified Mayo operation on forty feet; analysing post-operative pain, metatarsalgia, func tion of the metatarsophalyngeal joint, and also measures the reduction of the width of the forefoot. The previous relevan literature is also briefly reviewed.

### SCOLIOSIS IN CORNWALL

**D J Bracey, Truro**

A limited amount of scoliosis surgery is performed in Cornwall, though careful case selection has been necessary to keep complications to a minimum. Between 1980 and 1986 forty-two operations were performed. The majority of the curves were in the thoracic region with an average POP angle of 71°. The average correction was 53%. The complication rate has been low and there have been no neurological problems. The increased use of intersegmental spinal fixation has reduced the need for bone grafting and the use of post-operative immobilisation. Whilst this type of surgery is pre-ferably dealt with in regional centres, Cornwall's relative surgical isolation will make it desirable if this type of surgery continues in Truro.

